
JOURNAL INFORMATION
==============================
NLM Title Abbreviation: Access Microbiol
Journal ID: Access Microbiol
NLM Journal ID: 101746927
Journal ID: acmi

Access Microbiology

EISSN: 2516-8290
Publisher: Microbiology Society

ARTICLE INFORMATION
==============================
PMCID: PMC7472543
PMID: 32974555
Article version: 1
Subjects: Oral Abstract

Abstracts from the British Yeast Group Meeting 2019

Publication date: 2019
Electronic publication date: 2019 Nov 29
Volume: 1
Issue: 9
Electronic Location ID: 6
Copyright: © 2019 The Authors
Copyright year: 2019
License: This is an open-access article distributed under the terms of the Creative Commons Attribution License.
License URL: https://creativecommons.org/licenses/by/4.0/

==============================


The AP-2 endocytic adaptor complex in Candida albicans morphology and virulence

http://jmmcr.microbiologyresearch.org

Knafler Harriet *
Rooij Iwona Smaczynska-de
Ayscough Kathryn
University of Sheffield , Sheffield , United Kingdom
*Correspondence:Harriet Knafler, hcknafler1@sheffield.ac.uk

The chitin attenuator: Cross-talk between calcineurin and the cell wall salvage pathways prevents chitin overexpression and loss of fungal viability

http://jmmcr.microbiologyresearch.org

Dantas Alessandra da Silva *
Walker Louise A.
Gow Neil A.R.
University of Exeter , UK , undefined
University of Aberdeen , Aberdeen , United Kingdom
*Correspondence:Alessandra da Silva Dantas, a.da-silva-dantas@exeter.ac.uk

High-throughput growth and data with ‘impact’: Converting the Singer testing fleet into a lab

http://jmmcr.microbiologyresearch.org

Severn Oliver *
Singer Instruments , Roadwater , United Kingdom
*Correspondence:Oliver Severn, oliver@singerinstruments.com

Data-driven prediction of genetic interactions in Candida glabrata

http://jmmcr.microbiologyresearch.org

Invergo Brandon
Ames Ryan
Usher Jane *
University of Exeter , Exeter , United Kingdom
*Correspondence:Jane Usher, j.usher@exeter.ac.uk

Functional characterisation of novel oxidative stress protection proteins in the pathogenic yeast Candida glabrata

http://jmmcr.microbiologyresearch.org

Usher Jane *
University of Exeter , Exeter , United Kingdom
*Correspondence:Jane Usher, j.usher@exeter.ac.uk

Engineering synthetic mini-chromosomes to study chromosome segregation in budding yeast

http://jmmcr.microbiologyresearch.org

Jonsson Katarina *
Zulkower Valentin
Robertson Daniel
Cai Yizhi
Marston Adele
The University of Edinburgh , Edinburgh , United Kingdom
Manchester Institute of Biotechnology , Manchester , United Kingdom
*Correspondence:Katarina Jonsson, s1140622@sms.ed.ac.uk

Mec1ATM/ATR is a multifaceted protein kinase promoting cellular response to genotoxic stress and perturbation in protein homeostasis

http://jmmcr.microbiologyresearch.org

Corcoles-Saez Isaac
Dong Kangzhen
Waskiewicz Erik
Costanzo Michael
Boone Charles
Cha Rita *
Bangor University , Bangor , United Kingdom
Toronto University , Toronto , Canada
*Correspondence:Rita Cha, r.cha@bangor.ac.uk

Using Saccharomyces cerevisiae as a tool for mapping genetic variants underlying differences in response to anti-cancer agents

http://jmmcr.microbiologyresearch.org

Almayouf Salwa *
Barton David B.H.
Hu Yu
Foster Steven
Louis Ed J.
University of Leicester , Leicester , United Kingdom
Coventry University , Coventry , United Kingdom
*Correspondence:Salwa Almayouf, sa725@le.ac.uk

The BioScreening Facility at Newcastle University

http://jmmcr.microbiologyresearch.org

Banks Peter *
Newcastle University , UK , undefined
*Correspondence:Peter Banks, peter.banks@ncl.ac.uk

Fungal Transformers: Tracking a Moving Target

http://jmmcr.microbiologyresearch.org

Childers Delma *
Avelar Gabriela Mol
Bain Judith
Pradhan Arnab
Larcombe Daniel
Erwig Lars
Gow Neil
Brown Alistair
MRC Centre for Medical Mycology at University of Aberdeen , Aberdeen , United Kingdom
Galvanibio , Stevenage , United Kingdom
University of Exeter , Exeter , United Kingdom
*Correspondence:Delma Childers, delma.childers@abdn.ac.uk

Investigating a novel commensal strain of Candida famata within Daniorerio

http://jmmcr.microbiologyresearch.org

Jones Daniel *
Hagen Ferry
Donaldson Christopher
Rudman Jacob
Canedo Jaime
Johnston Simon
University of Sheffield , Sheffield , United Kingdom
Sheffield Hallam University , Sheffield , United Kingdom
Westerdijk Institute , Utrecht , Netherlands
*Correspondence:Daniel Jones, dwj945@gmail.com
Print publication date: 2019 Nov
Electronic publication date: 2019 Nov 29
Volume: 1
Issue: 9
Electronic Location ID: 44
Copyright: © 2019 The Authors
Copyright year: 2019
License: This is an open-access article distributed under the terms of the Creative Commons Attribution License.
License URL: http://creativecommons.org/licenses/by/4.0/

